# Home-based exercise and bone mineral density in peritoneal dialysis patients: a randomized pilot study

**DOI:** 10.1186/s12882-021-02289-y

**Published:** 2021-03-18

**Authors:** Kumi Watanabe, Yuka Kamijo, Mai Yanagi, Yoshitaka Ishibashi, Taku Harada, Masahiro Kohzuki

**Affiliations:** 1 Department of Nephrology, Japanese Red Cross Medical Center, 4-1-22 Hiroo, Shibuya, Tokyo, 150-8935 Japan; 2 Department of Internal Medicine and Rehabilitation Science, Tohoku University Graduate School of Medicine, 1-1 Seiryomachi, Aoba, Sendai, Miyagi 980-8574 Japan

**Keywords:** Osteoporosis, Physical function, Physical activity, Fall prevention

## Abstract

**Background:**

The prevalence of osteopenia and osteoporosis is higher in patients with chronic kidney disease than that in the general population. Although physical exercise prevents bone loss in hemodialysis (HD) patients, previous studies have not focused on peritoneal dialysis (PD) patients. Therefore, we aimed to evaluate the effects of home-based exercise on bone mineral density (BMD) in patients with PD.

**Methods:**

Stable outpatients undergoing PD were randomly assigned to the intervention group (*n* = 26; male, 20; median age, 66 years) or usual-care group (*n* = 27; male, 21; median age, 64 years). Patients in the intervention group performed home-based exercises (resistance exercise, stretching, and aerobic exercise such as walking) for 6 months, whereas those in the usual-care group performed stretching and their usual physical activity. Based on dual X-ray absorptiometry, the primary outcomes were the BMD data of the lumbar spine and proximal femoral neck. Secondary outcomes included physical function and physical activity. Pre- and post-intervention values were compared.

**Results:**

There was no significant within-group change in the BMD of the lumbar spine, femoral neck, and hip after 6 months of the exercise program. The intervention group had significantly improved 30-s chair-stand test, 6-min walk test, and physical activity results.

**Conclusions:**

Home-based exercises in patients with PD did not improve BMD at any of the sites evaluated. Improvement in physical function and physical activity may reduce the risk of falls in patients with PD.

**Trial registration:**

UMIN Clinical Trials Registry, UMIN000041678. Registered September 4, 2020; retrospectively registered.

**Supplementary Information:**

The online version contains supplementary material available at 10.1186/s12882-021-02289-y.

## Background

Osteoporosis and osteopenia are common complications in chronic kidney disease (CKD) patients [1]. The risk of bone fracture increases with the deterioration of kidney function [2]. The incidence rate of hip fractures in these patients is more than four times higher than the incidence in the general population [3]. Characteristically, these patients have CKD mineral and bone disorder (CKD-MBD) [4], which is influenced by uremic osteoporosis [5], aging [6], sarcopenia [7], and inactivity [8]. In addition, dialysis patients with fractures may have more difficulty performing daily activities [9] and may be at risk of morbidity and mortality [10].

In current practice, the safety and effectiveness of antiresorptive drugs for osteoporosis treatment are not clearly established [11] because of the possibility of side effects among patients with stage 3–5 CKD [12]. Among the helpful therapeutic recommendations for osteoporosis, exercise therapy has been regarded as an effective and easy intervention [13]. Several previous studies indicated that resistance or multicomponent exercises positively influence bone mineral density (BMD) and bone turnover in individuals without CKD [14–16].

In contrast, physical exercises have been shown to improve cardiovascular outcomes, physical function, dialysis efficiency, and health-related quality of life in dialysis patients [17]. Recently, several studies on hemodialysis (HD) patients have reported that resistance exercise can increase BMD [18]. Nevertheless, whether exercise affects BMD in peritoneal dialysis (PD) patients remains unclear. Furthermore, PD patients undergo dialysis mainly at their homes, and the frequency of attending the hospital is less than that of HD patients. Therefore, the exercise program may not be the same in HD patients who performed supervised intradialytic exercise for several hours of receiving treatments per week [19]. Hence, applying a more feasible method of home-based exercise is necessary.

In this study, we hypothesized that home-based exercise improves BMD. Therefore, this study aimed to examine the effects of a 6-month home-based exercise program on the BMD of patients with PD.

## Methods

### Subjects

Outpatients undergoing PD from the Japanese Red Cross Hospital (Tokyo, Japan) from October 2017 to December 2017 were recruited. PD modality was provided as continuous ambulatory PD (CAPD), automated PD (APD), and CAPD+APD. Combined therapy (PD + HD) was also provided to the patients. The study adheres to CONSORT guidelines (Additional file 1).

The inclusion criteria were PD therapy or combined therapy (PD + HD) for more than 3 months and willingness to undergo the study protocol. The exclusion criteria were unstable hypertension, recent myocardial infarction or unstable angina, congestive heart failure (grade > II according to the New York Heart Association Functional Classification), arthritic or orthopedic problems that limit exercise and require assistance during walking, cognitive disorders (Mini-Mental State Examination score < 24) [20], and change in hemodialysis type.

### Study design

The study was a stratified randomized pilot study, and 71 patients were included in the study after applying the inclusion and exclusion criteria. The patients were divided into the intervention and usual-care groups using computer-generated randomization, which stratified patients by sex, PD modality (PD or combined therapy [PD + HD]), and diabetic status (yes or no).

### Intervention program

The intervention group performed a home-based exercise for 6 months. Based on the ACSM’s guidelines [21], home-based exercises included walking, resistance exercises, and stretching. We requested that participants walk for 20–30 min, 3–5 times per week. and to increase to 500–1000 steps, depending on their ability, every month. Resistance exercises for the upper body, including shoulder press, biceps curl, and shoulder bench press in standing or sitting, were performed using TheraBand (The Hygenic Corporation, Akron, OH, USA). Lower body exercises included squats, calf raises, hip abductions, and unipedal standing. These exercises were started initially as one set of 10–15 repetitions and gradually increased up to the required number of sets using intensity-changing TheraBand. The intensity of aerobic and resistance exercises was a Borg scale score of 11–13 [22]. Stretching exercises were targeted at a large muscle group. During the exercise intervention, we advised the participants to be careful not to pull the peritoneal catheter and to attempt exercising with fluid in their abdomen; however, if this produces discomfort, they were encouraged to drain the fluid before exercising [21].

During their regular monthly hospital visit, patients performed home exercises under the supervision of a nurse to ensure compliance with the method, and lower aerobic exercise was performed using an ergometer (TERASUERUGO; Showa Denki Company Ltd., Osaka, Japan). To monitor adherence to the home-based exercise regimen, participants recorded the number of steps per day, repetition of resistance exercises, and frequency of exercise on a logbook with calendar-like pages. The patients brought this logbook during hospital visits for assessment.

Based on a previous study [23], we provided feedback and encouragement to the patients to continue exercising at home. In addition, we sent mails and called patients fortnightly to monitor their adherence to the home-based exercise program.

### Usual-care program

The usual-care group was assigned a program that focused on well-being. These patients received instructions on stretching the upper and lower body (low-frequency, low-intensity) for 6 months and were asked to carry on as usual.

### Primary outcome measure

#### Bone mineral density

BMD was measured using dual-energy X-ray absorptiometry (Horizon X, Discovery 20R series; Hologic, Marlborough, MA, USA) at the lumbar spine (L2–L4) and left proximal femur (femoral neck and hip). The bone T-score, which is the number of standard deviations greater than or less than the mean BMD of normal young adults according to the definitions of the World Health Organization, was calculated for the lumbar spine and proximal femur in each patient.

### Secondary outcome measures

#### Handgrip force dynamometry

Handgrip force is an assessment of muscle function [24] measured using a dynamometer (T.T.K. 5401 GRIP D; Takei Science Instruments, Niigata, Japan). During measurement, patients stood upright with their arms hanging on their sides, and the dynamometer was placed close to their bodies. They were asked to exert maximum force on the dynamometer twice using the left and right arms alternately. The highest average value was recorded in kilograms.

#### 30-s chair-stand test

The 30-s chair-stand test (CS-30) was performed to evaluate lower extremity muscle strength [25]. A standardized chair with a seat height of 40 cm was used. Patients were asked to stand up fully and sit down as many times as possible within 30 s with their arms across their chests. The score was the total number of stands executed correctly, and a zero score was recorded if the patient was unable to rise from the chair without using the arms.

#### 6-minute walk test

For this study, the 6-min walk test (6MWT) was performed according to the recommendations of the American Thoracic Society [26]. The 6MWT was conducted in a 30-m corridor. Patients were required to walk the longest distance as possible in 6 min by walking 30 m and then going back. They could stop if needed and restart the test.

#### Physical activity

A three-axis accelerometer (AM500N; Accores Company Limited, Nagano, Japan) was used to measure patients’ daily physical activity, which includes the number of steps (steps/day), number of moderate-intensity steps (steps/day), and energy expenditure (determined by the calculated METs with daily activity [kcal/day]). All patients agreed to wear an accelerometer for 6 months, except during sleep and bathing. Patients were instructed to wear the device around their waist. The recorded physical activity data were downloaded once a month during outpatient visits, and the average of the 7-day physical activity data was recorded [27].

### Statistical analysis

This study was analyzed using a per-protocol (intervention group, 26; usual-care group, 27). Continuous data were expressed as mean ± standard deviation and were analyzed using the paired t-test, whereas categorical data were expressed as frequencies (%) and were evaluated using the chi-square test. For the analysis of BMD between baseline and 6 months, a two-way repeated-measures analysis of variance was applied within the group. Within-group change (% change = [post-value-pre-value]/pre-value× 100) between baseline BMD (pre) and 6-month BMD (post) was calculated, and an independent t-test was performed.

For the analysis of the second outcomes between baseline and end of the intervention, a two-way repeated-measures analysis of variance was applied, with group and time as predictor variables. Within-group analysis among baseline, 3 months, and 6 months was performed using one-way analysis of variance. The Bonferroni test was used to determine significant differences. We conducted two nonparametric tests: the Friedman test to check for significant differences among baseline, 3 months, and 6 months, and the Wilcoxon rank sum test between the two baseline values. Statistical significance was accepted at *p* < 0.05, and statistical analyses were performed using EZR version 1.37 (http://www.jichii.ac.jp/saitama-sct/SaitamaHP.files/statmed.html) [28].

## Results

### Study participants

**Fig. 1:**
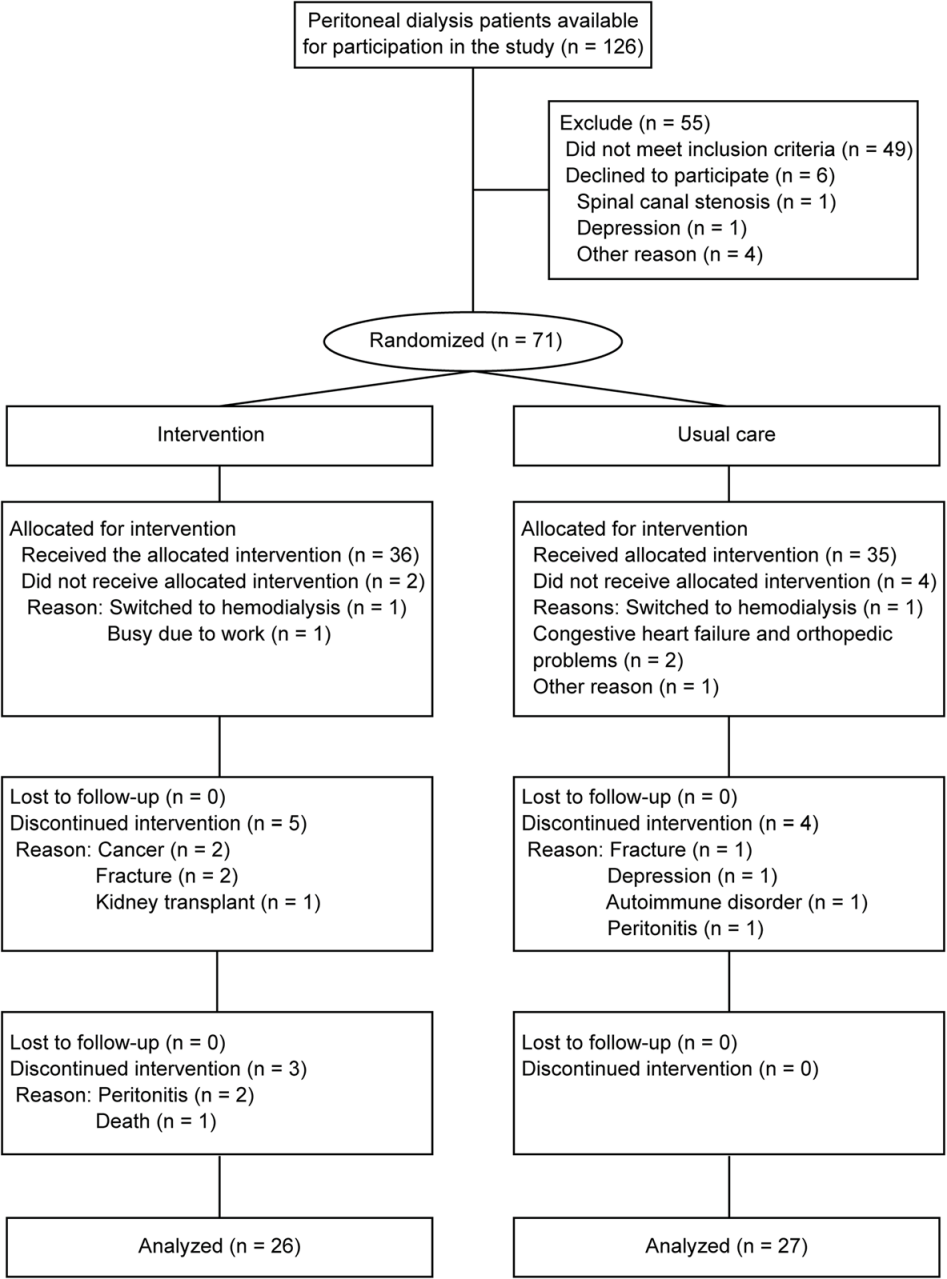
Flowchart of the participants

Among the 126 patients screened, 55 patients were excluded because they did not meet the eligibility criteria or declined to participate (Fig. 1), and 71 patients were randomized into the intervention and usual-care groups. Two patients from the intervention group and four patients from the usual-care group did not receive the allocated intervention. Thus, a total of 65 patients were enrolled; however, only 53 (82%) patients completed the study. The baseline characteristics are shown in Table 1. No significant differences in baseline characteristics were found between the groups.

**Table 1:** Baseline patient characteristics

	Intervention (***n*** = 26)	Usual care (***n*** = 27)	***p***
Male sex	20 (76.9)	21 (77.8)	1.00
Age (years)	66.19 ± 13.05	64.00 ± 12.95	0.542
PD vintage (years)	5.04 ± 3.10	5.07 ± 3.92	0.971
PD modality			0.428
CAPD	15 (57.5)	16 (59.3)	
APD	9 (34.6)	7 (25.9)	
CAPD+APD	2 (7.7)	4 (14.8)	
HD + PD combined therapy	16 (61.5)	13 (48.1)	0.412
BMI (kg/m^2^)	22.52 ± 3.94	23.30 ± 4.55	0.505
Kt/V PD	1.92 ± 0.47	1.85 ± 0.43	0.555
DM	8 (30.8)	9 (33.3)	1.00
Calcium (mg/dL)	8.51 ± 0.73	8.63 ± 0.77	0.562
Phosphates (mg/dL)	6.40 ± 2.01	5.73 ± 1.29	0.151
Albumin (g/dL)	3.44 ± 0.50	3.44 ± 0.32	0.995
Prealbumin (mg/dL)	39.22 ± 10.12	37.18 ± 8.44	0.432
Total protein (g/dL)	6.25 ± 0.78	6.43 ± 0.44	0.297
HbA1c (%)	5.74 ± 0.58	5.96 ± 0.70	0.226
Hemoglobin (g/dL)	11.44 ± 1.81	11.81 ± 1.53	0.423
Fasting blood sugar (mg/dL)	122.71 ± 46.81	116.68 ± 32.16	0.586
Cause of ESRD			
Diabetes	8 (30.8)	7 (25.9)	0.766
Glomerulonephritis	8 (29.6)	8 (30.8)	1.00
Nephrosclerosis	1 (3.8)	3 (11.1)	0.61
IgA nephropathy	5 (19.2)	1 (3.7)	0.1
Other diseases	4 (15.4)	8 (29.6)	0.327
Medication of CKD-MBD			
Phosphorus adsorbent	23 (88.5)	24 (88.9)	1.00
Calcium receptor	14 (53.8)	10 (37.0)	0.275
Active vitamin D3	11 (42.3)	16 (59.3)	0.276

Values are given as mean ± standard deviation or n (%)

Continuous variables, Student’s t-test; categorical data, chi-square test

*PD* peritoneal dialysis, *CAPD* continuous ambulatory peritoneal dialysis, *APD* ambulatory peritoneal dialysis, *HD* hemodialysis, *BMI* body mass index, *K* dialyzer clearance of urea, *t* dialysis time, *V* volume of distribution of urea, *DM* diabetes mellitus, *HbA1c* glycated hemoglobin, *ESRD* end-stage renal disease, *IgA* immunoglobulin A, *CKD-MBD* chronic kidney disease-mineral and bone disorder

### Primary outcome

#### Bone mineral density

**Fig. 2:**
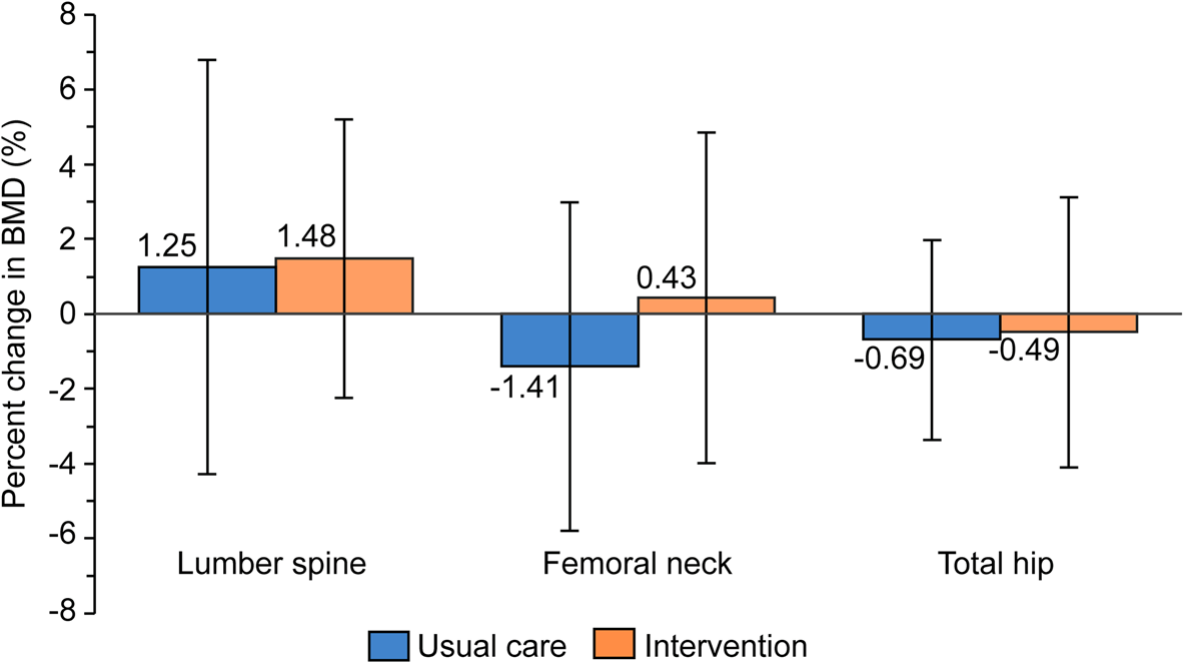
Percentage of change in bone mineral density (BMD) after 6 months of exercise program

Pre- and post-study values for BMD and T-scores are shown in Table 2. There was no significant interaction in BMD at any of the site evaluated after the 6-month exercise program. Figure 2 shows the within-group change in BMD. There was no significant within-group change in BMD at any of the sites after the 6-month exercise program.

**Table 2:** Changes in bone mineral density (g/cm^2^)

	Intervention		Usual care		Group	Time	Group×Time
	Baseline	6 months	Baseline	6 months			
Lumbar (L2–L4)	0.958 ± 0.182	0.995 ± 0.188*	0.991 ± 0.195	1.039 ± 0.209	0.42	0.01	0.785
Lumbar (L2–L4) T-score	−0.652 ± 1.459	0.362 ± 1.467*	−0.423 ± 1.46	−0.077 ± 1.554	0.47	0.02	0.796
Neck	0.611 ± 0.127	0.639 ± 0.122	0.648 ± 0.141	0.661 ± 0.145	0.48	0.49	0.179
Neck T-score	−1.905 ± 0.989	−1.692 ± 0.953	−1.606 ± 1.070	−1.507 ± 1.128	0.45	0.57	0.256
Total hip	0.720 ± 0.147	0.741 ± 0.122	0.761 ± 0.164	0.779 ± 0.165	0.40	0.11	0.751
Total hip T-score	−1.688 ± 1.088	−1.551 ± 0.889	−1.387 ± 1.169	−1.266 ± 1.187	0.37	0.09	0.854

Values are given as mean ± standard deviation

Group×Time is an interaction from a two-way repeated-measures analysis of the effect of time from baseline to 6 months

*p < 0.05 vs. baseline within-group difference

### Secondary outcome measure

#### Physical function

**Fig. 3:**
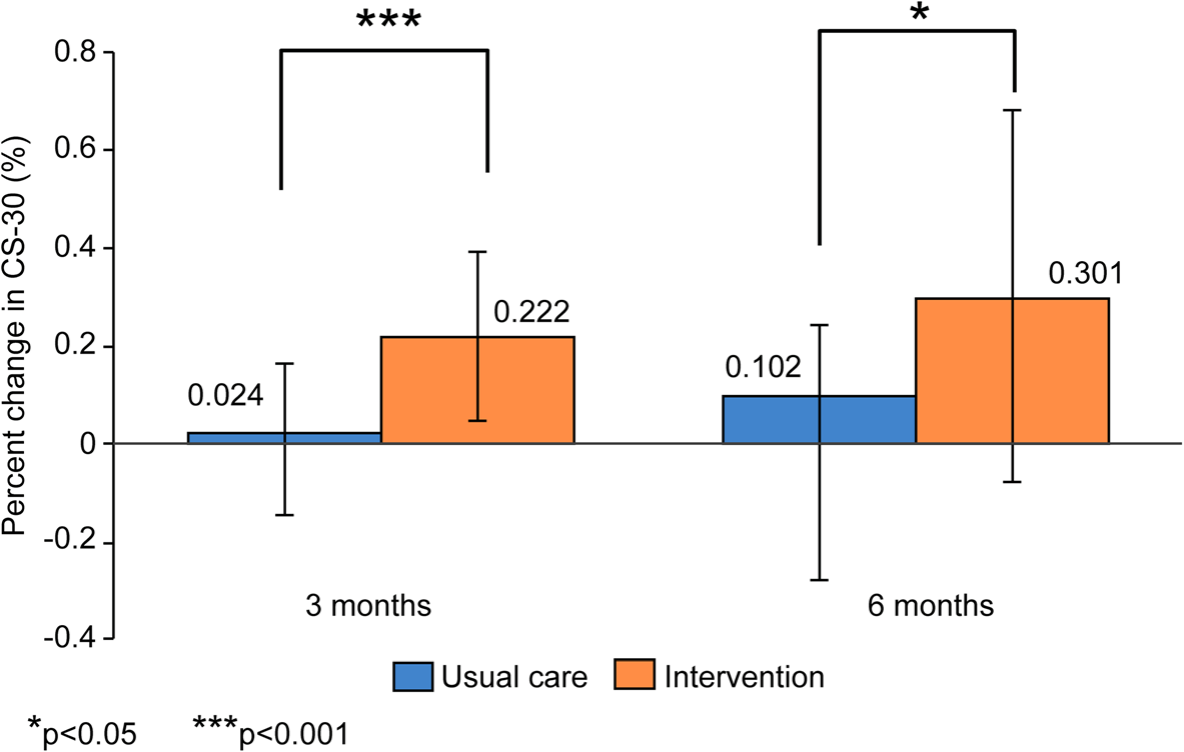
Percentage of change in the 30-s chair-stand test (CS-30) after 3 and 6 months of exercise program

**Fig. 4:**
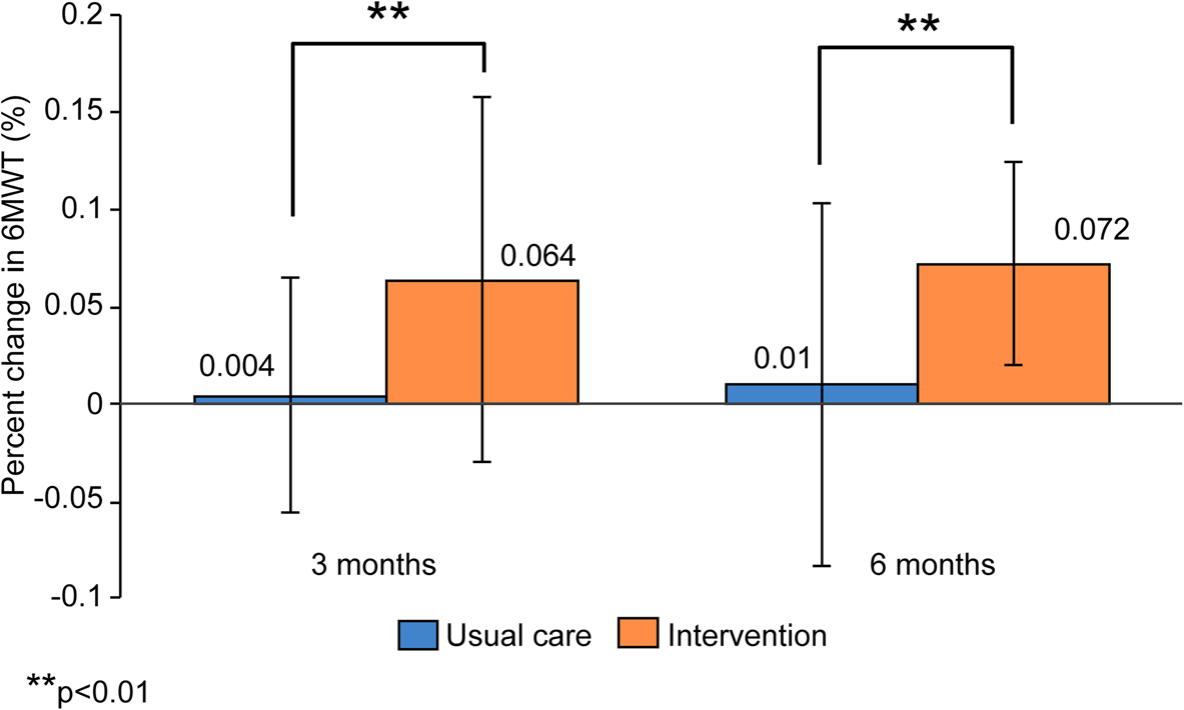
Percentage of change in the 6-min walk test (6MWT) after 3 and 6 months of exercise program

The results of the physical function assessments are shown in Table 3. For the intervention group, the results of the CS-30 and 6MWT increased significantly at 3 and 6 months compared with baseline levels. In addition, a significant group×time interaction was observed for CS-30 (*p* < 0.001) and 6MWT (*p* < 0.01). Figure 3 shows the within-group change at 3 (*p* < 0.001) and 6 months (*p* < 0.05) in CS-30. Figure 4 shows the within-group change at 3 (*p* < 0.001) and 6 months (*p* < 0.001) in the 6MWT.

**Table 3:** Change in physical function and physical activity

	Intervention			Usual care			Group	Time	Group ×Time
	Baseline	3 months	6 months	Baseline	3 months	6 months			
Grip strength (kg)	28.7 ± 9.7	28.1 ± 10.4	28.9 ± 9.7	27.8 ± 8.1	27.1 ± 8.1	27.1 ± 8.5	0.75	0.27	0.66
CS-30 (rep)	12.5 ± 3.7	15.2 ± 3.7***	17.0 ± 4.7***†††	13.6 ± 4.2	13.7 ± 3.6	14.8 ± 4.4**†	0.45	< 0.001	< 0.001
6MWT (m)	427.1 ± 84.9	453.9 ± 96.4***	461.0 ± 95.3***	445.5 ± 96.9	447.6 ± 99.9	448.8 ± 100.7	0.99	< 0.001	< 0.01
Average daily number of steps (steps/day)	4820.5 ± 2698.6	5710.7 ± 2698.8	5316.8 ± 2254.5	5817.9 ± 3761.7	5032.5 ± 3177.4	5011.3 ± 3679.7	0.89	0.29	0.009
Average daily number of moderate steps (steps/day)	3577.1 ± 1762.8	3959.4 ± 1919.8	4130.5 ± 2236.3	4225.7 ± 3089.1	3702.8 ± 2671.8	3793.8 ± 3218.6*	0.96	0.99	0.014
Average daily energy expenditure (kcal)	255.7 ± 86.5	278.4 ± 97.9	282.2 ± 106.8	314.1 ± 157.2	263.1 ± 149.9**	259.6 ± 184.2**	0.96	0.47	0.002
Average daily moderate activity time (minutes/day)	37.9 ± 15.8	42 ± 19.2	43.3 ± 20.4	47.4 ± 32.1	41.7 ± 29.3	42.8 ± 35.5	0.82	0.94	0.014

Values are given as mean ± standard deviation

Group×Time is an interaction from a two-way repeated-measures analysis of the effect of time from baseline to 6 months

*CS-30* 30-s chair-stand test, *rep* repetitions, *6MWT* 6-min walk test

*p < 0.05 vs. baseline within-group difference

**p < 0.01 vs. baseline within-group difference

***p < 0.001 vs. baseline within-group difference

†*p* < 0.05 vs. month 3 within-group difference

†††*p* < 0.001 vs. month 3 within-group difference

#### Physical activity

The results of the physical activity assessment are shown in Table 3. A significant group×time interaction was observed for the average daily number of steps (*p* < 0.009), number of moderate steps (*p* < 0.014), energy expenditure (*p* < 0.002), and moderate activity time (*p* < 0.014). In the usual-care group, the number of steps and energy expenditure were significantly decreased. In the intervention group, the Friedman test among baseline, 3 months, and 6 months showed significant differences in the number of steps (*p* < 0.05), average daily energy expenditure (*p* < 0.01), and average daily moderate activity time (*p* < 0.05). The number of moderate-intensity steps showed no significant difference. In the control group, these variables showed no significant difference. In addition, the Wilcoxon rank sum test between the baseline values of the control and intervention groups showed no significant difference.

#### Adverse events and adherence to exercise program

No adverse events directly related to exercise training during the 6-month study period were reported. From the medical records, fracture (three patients) and peritonitis (three patients) were not attributed to the intervention exercise (Fig. 1).

## Discussion

We assessed the effect of a 6-month home-based exercise program on the BMD of patients with PD. The exercise protocols resulted in significant improvement in physical function and physical activity in the intervention group compared with the usual-care group. Our hypothesis that a home-based exercise program would improve BMD within 6 months was not supported.

Many systematic review and meta-analysis have reported that an exercise intervention can increase BMD in both the lumbar spine and femoral neck [14–16]. In postmenopausal women, combined resistance exercise and high-impact or weight-bearing training improved BMD [14]. In older adults, a similar combined exercise prevented bone loss or increased BMD [16]. Additionally, in middle-aged and older men, resistance exercise alone or a combination of resistance exercise and high-impact loading activity might help prevent osteoporosis [15].

After a 6-month exercise program, there was no significant change in BMD at any of the sites within the groups. It remains possible that the intensity of resistance exercise was lower than that reported in previous studies [14–16]. Liao et al. reported that intradialytic aerobic cycling exercise for 3 months contributed to a negligible loss of BMD in the femoral neck after 1 year [29]. Intradialytic aerobic cycling might add resistance, in contrast to home-based exercises, which have a low load on the bone.

Many previous reviews have shown that an exercise regimen of 12 months or longer is effective in improving BMD [14–16] because of the length of the remodeling cycle [30]. Therefore, we should consider the evaluation of BMD from exercise from a long-term perspective.

We found that physical function, as determined by CS-30 and 6MWT, improved in the intervention group. A previous study showed that improved fitness and muscle strength contribute to the prevention of falls and a lower risk of fracture [31]. Fall prevention may be an important method of protecting patients with end-stage renal disease from hip fractures [32]. Kawabata and Hiura reported that the cutoff value for CS-30 was 14.5 repetitions in the healthy Japanese elderly population [33]. The finding of an increase from 12.5 to 17.0 repetitions in the intervention group suggested that it was useful for fall prevention. Although there was significant difference in the usual-care group at 6 months, we considered the possibility of the Hawthorne effect on the basis of the percentage of change and minimum clinically significant differences in the CS-30.

Physical activity tended to increase in the intervention group but decreased significantly in the usual-care group. The pedometer-determined number of steps per day was positively associated with the total hip BMD community-based elderly patients [34]. Similarly, Rodríguez-Gómez et al. reported that older people might have decreased bone fracture risk through physical activity increase and sedentary behavior reduction [35]. In contrast, vigorous and moderate physical activities were not associated with bone density and surrogate markers of CKD-MBD in HD patients [36]. Although whether physical activity has an influence on BMD was unclear, there are concerns that the level of physical activity in CKD patients decreases with disease progression [37]; furthermore, aging is associated with bone loss. Physical inactivity is a risk factor for mortality in patients with CKD [38]; thus, it is necessary to continuously improve physical activity habits among these patients.

This study had several limitations. First, the intensity of resistance exercise may be lower than that of supervised intradialytic exercise in HD patients because PD patients performed the exercise program at home. High-intensity exercise was more effective than low-intensity exercise in improving BMD [14–16]. Konstantinidou et al. reported that training at home could not be supervised at all; therefore, it was difficult to ensure compliance [39]. Second, the intervention period was determined by considering the participants’ adherence to the home-exercise regimen. However, the 6-month intervention period may have been shortened to improve BMD. Third, the sample size was small and the participants included not only typical PD patients but also those undergoing combined HD and PD; therefore, the generalizability of the study findings was weak. By increasing the number of participants/sample size in future studies, the possibility of type 2 statistical error will decline. Finally, our analysis in this RCT studies had an important limitation: there was attrition bias in the per-protocol analysis that excluded patients who deviated from the protocol.

## Conclusions

The results of our study suggest that home-based exercises for 6 months in patients with PD do not contribute to the maintenance or improvement of BMD. However, improvement in physical function and physical activity may reduce the risk of falls in patients with PD. Additional studies with exercise protocols involving regular intensity exercise and longer intervention periods are warranted to evaluate the effects of the exercise.

## Supplementary Information

**Additional file 1:.** CONSORT checklist.

## Data Availability

The datasets used and/or analyzed during the current study are available from the corresponding author upon reasonable request.

## Abbreviations

PD: Peritoneal dialysis
HD: Hemodialysis
CKD: Chronic kidney disease
BMD: Bone mineral density
